# Clinically Relevant Radiation Exposure Differentially Impacts Forms of Cell Death in Human Cells of the Innate and Adaptive Immune System

**DOI:** 10.3390/ijms19113574

**Published:** 2018-11-13

**Authors:** Sylvia E. Falcke, Paul F. Rühle, Lisa Deloch, Rainer Fietkau, Benjamin Frey, Udo S. Gaipl

**Affiliations:** Department of Radiation Oncology, Universitätsklinikum Erlangen, Friedrich-Alexander-Universität Erlangen-Nürnberg, 91054 Erlangen, Germany; sylvia_f@gmx.de (S.E.F.); paul-friedrich.ruehle@a-b-f.de (P.F.R.); lisa.deloch@uk-erlangen.de (L.D.); rainer.fietkau@uk-erlangen.de (R.F.); benjamin.frey@uk-erlangen.de (B.F.)

**Keywords:** human peripheral blood immune cells, radiosensitivity, forms of cell death, high-dose radiotherapy (HDRT), low-dose radiotherapy (LDRT), radiation protection

## Abstract

In cancer treatments, especially high-dose radiotherapy (HDRT) is applied. Patients suffering from chronic inflammatory diseases benefit from low-dose radiation therapy (LDRT), but exposure to very low radiation doses can still steadily increase for diagnostic purposes. Yet, little is known about how radiation impacts on forms of cell death in human immune cells. In this study, the radiosensitivity of human immune cells of the peripheral blood was examined in a dose range from 0.01 to 60 Gy with regard to induction of apoptosis, primary necrosis, and secondary necrosis. Results showed that immune cells differed in their radiosensitivity, with monocytes being the most radioresistant. T cells mainly died by necrosis and were moderately radiosensitive. This was followed by B and natural killer (NK) cells, which died mainly by apoptosis. X-radiation had no impact on cell death in immune cells at very low doses (≤0.1 Gy). Radiation doses of LDRT (0.3–0.7 Gy) impacted on the more radiosensitive NK and B cells, which might contribute to attenuation of inflammation. Even single doses applied during RT of tumors did not erase the immune cells completely. These in vitro studies can be considered as the basis to optimize individual radiation therapy schemes in multimodal settings and to define suited time points for further inclusion of immunotherapies.

## 1. Introduction

Radiotherapy (RT) plays an important role in modern medicine for treatment of both malign and benign diseases. Most often, external X-rays are applied. RT can be divided into high-dose RT (HDRT: single dose >1.0 Gy) and low-dose RT (LDRT: single dose ≤1.0 Gy). HDRT is utilized in cancer therapies. Generally, single doses per fraction of 1.8–2.0 Gy are used in norm-fractionated RT, and a total dose of up to 70 Gy is delivered depending on tumor entity and location.

Today, about 60% of all anticancer therapies include a HDRT, with the main aim being to achieve local tumor control [1]. In addition to killing tumor cells by radiation, immune modulations by RT are also observed, which might result in specific antitumor immune responses (summarized in References [2,3]).

LDRT comprises single doses of 0.3–1.0 Gy (cumulative dose: 3–6 Gy) for treatment of benign syndromes, such as chronic painful degenerative and inflammatory diseases. In Germany, 50,000 patients are treated with LDRT annually [4]. With this therapy, amelioration of inflammation combined with long-term pain reduction is observed [5,6]. The osteoimmunological modes of action of LDRT are the main mechanistic bases for this effect [7,8].

Yet, little is known about how radiation impacts on immune cells even though they are present in the tumor and its microenvironment as well as in inflamed and degenerated areas. Detailed immunophenotyping is increasingly being applied for definition of prognostic and predictive markers for inflammatory and cancer diseases [9,10,11]. However, it has to be taken into account that RT might not only result in immune activation but also in immune suppression (summarized in Reference [12]). The latter includes killing of immune cells.

Ionizing radiation can either directly or indirectly (via reactive oxygen species (ROS)) induce DNA double strand breaks (DSB). The repair of these complex damages is often error-prone, incomplete, or even impossible (summarized in Reference [13]). In addition, cells might be affected by irradiated cells in close proximity even though they were not hit themselves. Those nontargeted or bystander effects are triggered by direct cell–cell communications or the release of signal molecules [14,15].

The sensing of radiation-induced DNA damage normally leads to an arrest in the cell cycle. Depending on the severity of the damage, it can either be repaired or the cell undergoes apoptosis. The latter is an active and highly regulated process, which results in fragmentation of cellular DNA followed by removal of the dying cell through scavenger cells such as macrophages. If the clearance fails, apoptotic cells rest in the body and may lose their membrane integrity at any time point of apoptosis. They are then called secondary necrotic cells, which activate the immune system (summarized in Reference [16,17]). Secondary necrotic cells are often called late apoptotic cells. This naming is more appropriate from an immunological point of view due to their immune activation properties, which is common in all necrotic cells that have disturbed membrane integrity [18]. One has to mention that cells with subG1 DNA content are either apoptotic or secondary necrotic. If cells get severely damaged, this might also lead to primary necrotic cell death. In contrast to apoptosis, no fragmentation of the DNA occurs, but the cell swells until it loses its integrity. It has become evident that in addition to apoptosis, even necrosis can be a programmed process and that various cell death pathways are triggered by DNA damage (summarized in References [19,20,21]).

Various investigations have demonstrated that immune cells specifically invade the irradiation field [22,23]. If the immune cells are hit by radiation when they are within the irradiation field, they might be killed. However, as immune cells are in circulation, local RT therapies do not destroy all immune cells, and the resting ones often preserve their function [24,25]. It has to be stressed that the sensitivity of immune cells toward radiation has been mostly determined by Comet Assay, which visualizes DNA damage in individual cells. A separation between apoptotic and secondary necrotic cells is not possible. Furthermore, in 2002, McLean and colleagues provided evidence that the Comet Assay measured lower apoptosis at all doses when compared to cell death detection by flow cytometry after staining of the cells with AnnexinV and propidium iodide (PI) [26]. Therefore, analyses by flow cytometry should be performed when a detailed impact of radiation on cell death in immune cells is sought. In vivo detailed analyses of the radiosensitivity of splenocytes of whole-body irradiated mice have already been carried out [27]. However, there has not been any research on the distinction between apoptosis and necrosis induction by radiation. The existing data on radiosensitivity of human cells are mostly based on survival curve analyses [28].

**Figure 1:**
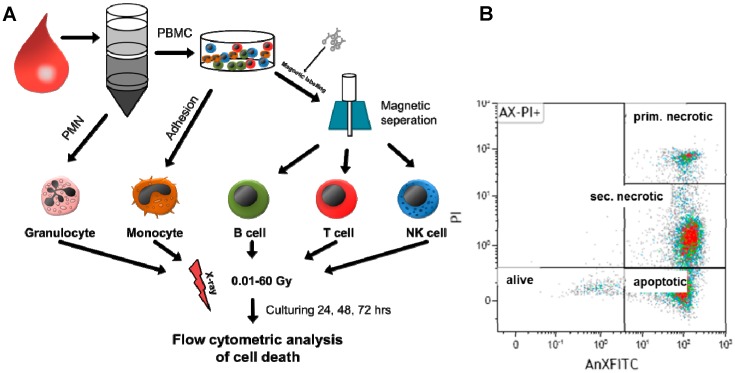
Scheme of experimental design showing the procedure of cell isolation and irradiation as well as cell death analysis by flow cytometry. (**A**) From whole-blood samples, various human immune cells were isolated by Ficoll separation followed by magnetic labeling or adherence techniques. Afterwards, cells were irradiated with X-rays (0.01–60 Gy). After 24, 48, or 72 h, cells were harvested and stained with AnnexinV (fluorescein isothiocyanate (FITC)-labeled) and propidium iodide (PI). (**B**) Then, samples were analyzed by flow cytometry for identification of viable (alive), apoptotic, primary (prim.) necrotic, and secondary (sec.) necrotic cells. The colors represent the amount of cells: high cell density: red; lower cell density: green.

The novelty of the present report is therefore the focused analyses of the impact of ionizing radiation over a wide dose range on forms of cell death in peripheral human immune cells. For this, various types of immune cells from healthy persons were isolated from the peripheral blood and subsequently irradiated with different single doses of X-rays. The studied dose range can be roughly divided into three categories: diagnostic radiation (≤0.1 Gy), LDRT applied for benign inflammatory diseases (0.3–1.0 Gy), and HDRT for malign tumors (>1 Gy). This is shown in Figure 1A. The amount of immune cells undergoing apoptosis or necrosis was determined by flow cytometry with Annexin V-FITC (fluorescein isothiocyanate)/PI staining (AxPI staining). AnnexinV specifically binds to phosphatidylserine, which is not present on the surface of living cells but is accumulated in the outer leaflet of apoptotic cells. PI intercalates into the DNA but only when the integrity of the cell membrane is disrupted. Consequently, a clear differentiation of living cells from apoptotic and necrotic ones was possible with this method (Figure 1B). Secondary necrotic cells could be differentiated from primary necrotic ones by reduced DNA content as these cells had already undergone apoptosis to a certain stage, including DNA fragmentation and consecutive exporting of DNA fragments via apoptotic blebs.

## 2. Results

### 2.1. Radiosensitivity of Peripheral Blood Lymphoid Cells (PBL) as Detected by SubG1 DNA Content and Analysis of Forms of Cell Death

**Figure 2:**
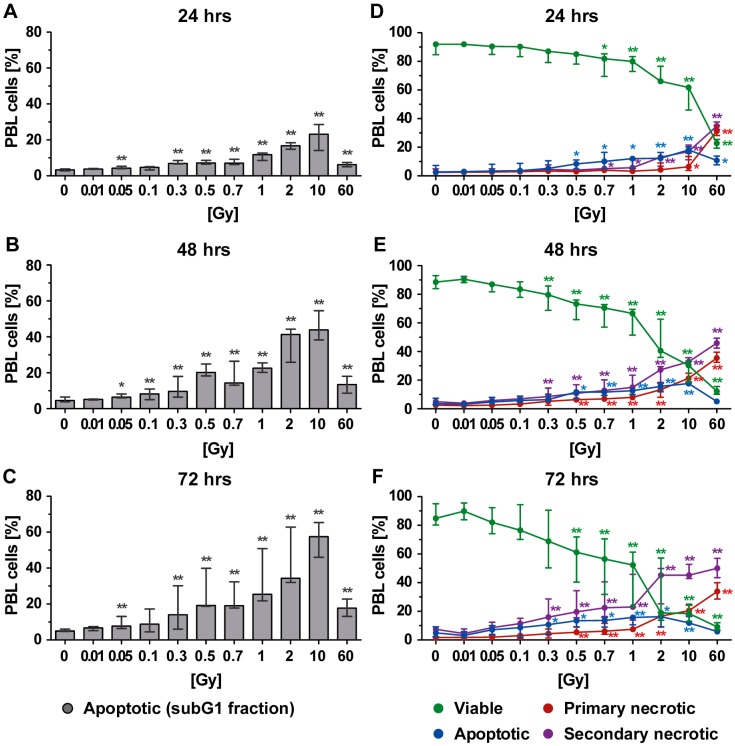
Cell death in peripheral blood lymphoid cells (PBL) at different time points after irradiation as detected by analyses of (**A**–**C**) subG1 DNA content and (**D**–**F**) phosphatidylserine exposure and membrane permeability of PI with AxPI staining. Each data point represents the median (±interquartile range (IQR)) from six experiments from three different donors. Data points have been connected by lines to improve visual clarity. Statistical analyses were performed against the corresponding nonirradiated control (0 Gy) using the Mann–Whitney *U* test (* *p* < 0.05; ** *p* < 0.01).

Peripheral blood lymphoid cells (PBL), which comprise T, B, and NK cells, were initially jointly analyzed to get the first indications about the radiosensitivity of key immune cells of the peripheral blood (Figure 2). Initially, the subG1 DNA content, which represents apoptotic and secondary necrotic cells, was determined by staining the PBL with PI in the presence of the detergent triton (PIT staining). This analysis revealed a dose-dependent increase in cells with subG1 DNA content (Figure 2A–C) after just 24 h of irradiation and starting from a dose of 0.05 Gy (Figure 2A). Similar results were observed 48 and 72 h after irradiation, but the percentage of cells with subG1 DNA content was further increased (Figure 2B,C). As this time-dependent increase was not detected in the nonirradiated control samples (Figure 2A–C: 0 Gy), it was directly linked to irradiation.

It must be noted that the amount of cells with subG1 DNA content appeared to decrease after exposure to the very high single dose of 60 Gy. This would suggest the existence of further types of cell death that could not be detected by subG1 DNA content analysis. Consequently, AxPI staining was additionally performed, which allowed us to distinguish between apoptosis, primary necrosis, and secondary necrosis (Figure 1B). This revealed that besides apoptosis, secondary necrosis was also present after radiation exposure. A dose-dependent increase in secondary necrosis was observed for all time points (Figure 2D–F: violet points). Likewise, an increase in primary necrotic cells was seen, especially after exposure of the PBL to a higher single dose of irradiation (≥2 Gy). Below 1 Gy, primary necrosis merely contributed to the death of PBL. As already described for the percentage of cells with subG1 DNA content, a decrease in apoptosis but an increase in necrosis was observed when PBL was irradiated with 10 or 60 Gy.

### 2.2. Forms of Cell Death in T Cells Following Radiation Exposure

**Figure 3:**
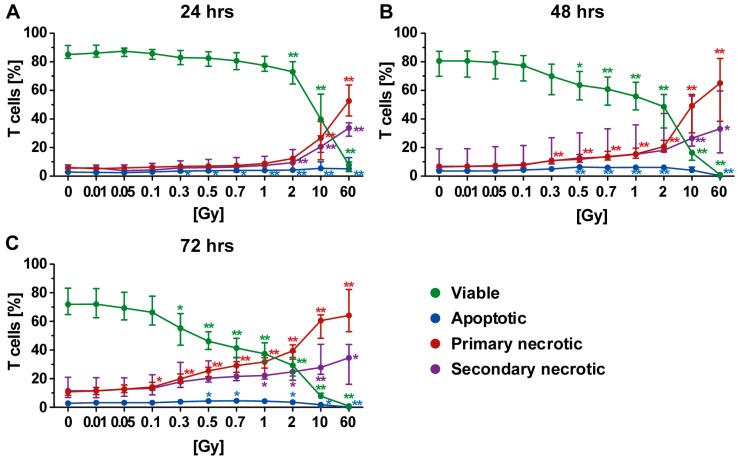
Forms of cell death in T cells at different time points after irradiation. (**A**–**C**) A radiation dose-dependent decrease in viable T cells (green) was observed. In particular, steady increases in primary (red) and secondary necrosis (violet) were identified to be linked to radiation dose. In contrast, the apoptosis rate (blue) seemed only to be marginally affected by radiation, suggesting that the T cells rapidly undergo secondary necrosis. (**A**–**C**) The colored dots represent the percentage distribution of viable (green), apoptotic (blue), primary (red), or secondary necrotic (violet) T cells as determined by AxPI staining and flow cytometry analyses at (**A**) 24, (**B**) 48, or (**C**) 72 h after irradiation. Each data point represents the median (±IQR) from six independent experiments from three different donors. Data points have been connected by lines to improve visual clarity. Statistical analyses were performed against the corresponding nonirradiated control (0 Gy) using the Mann–Whitney *U* test (* *p* < 0.05; ** *p* < 0.01).

We then examined the radiosensitivity of T, B, and NK cells separately. T cells represent about 60–70% of the cell population of PBL. Most of the dying T cells following radiation exposure were primary necrotic ones (Figure 3). Twenty-four hours post irradiation, the T cells were scarcely influenced in their viability by radiation with a dose below 2 Gy (Figure 3A: green line). However, the viability of T cells decreased at later time points after exposure to lower single doses of radiation (48 h: ≥0.5 Gy or 72 h: ≥0.3 Gy; Figure 3B,C).

In general, the percentage of apoptotic T cells was low, although a tiny increase was identified following irradiation with 0.5 Gy or more. However, as already observed for PBL (Figure 2), a decrease in apoptosis was detected following irradiation with higher doses (10 or 60 Gy). Here, T cells died via necrosis. When investigating later time points after irradiation exposure, both necrosis forms were significantly increased starting from a dose of 0.1 Gy. However, even 72 h after irradiation with 2 Gy, more than 30% of all T cells were still viable (Figure 3C).

Culturing conditions only slightly contributed to death of T cells, indicating that irradiation was the prominent stress factor. Irradiation with a dose between 0.3 and 2.0 Gy especially resulted in elevated percentage of dead T cells, which increased depending on both radiation dose and time after irradiation.

### 2.3. Forms of Cell Death in B Cells Following Radiation Exposure

**Figure 4:**
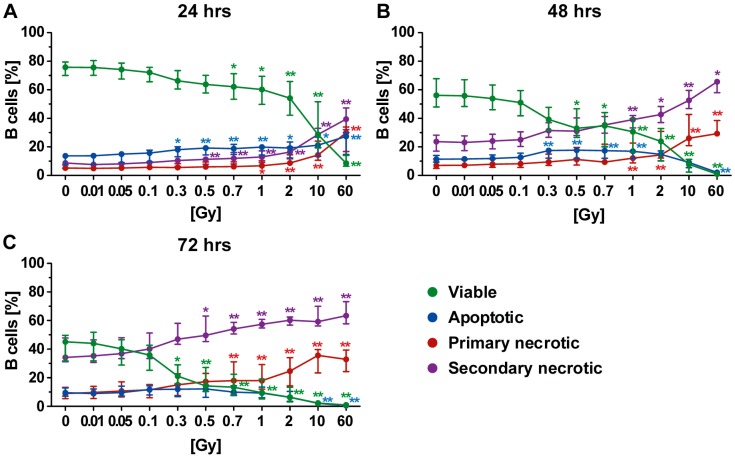
Forms of cell death in B cells at different time points after irradiation. (**A**–**C**) A high rate of secondary necrotic B cells (violet) was observed, which was time- and radiation-dependent, while low amounts of apoptotic cells were present (blue). Furthermore, a radiation-dependent increase in primary necrotic cells (red) was identified. The colored dots represent the percentage distribution of viable (green), apoptotic (blue), primary (red), or secondary necrotic (violet) B cells as determined by AxPI staining and flow cytometry at (**A**) 24, (**B**) 48, or (**C**) 72 h after irradiation. Each data point represents the median (±IQR) from ten independent experiments of five different donors. Data points have been connected by lines to improve visual clarity. Statistical analyses were performed against the corresponding nonirradiated control (0 Gy) using the Mann–Whitney *U* test (* *p* < 0.05; ** *p* < 0.01).

The B cells represent a minor part of PBL and showed partially varying results compared to T cells. Similarly, a radiation dose-dependent killing was revealed, but culturing conditions here strongly contributed to lowered survival rates of the B cells. Yet, irradiation still remained the prominent stress factor (Figure 4A–C). Following irradiation, the most prominent type of cell death was secondary necrosis (Figure 4B,C). Furthermore, a radiation dose-dependent increase in primary necrosis was observed, particularly after exposure of B cells to a single dose of 2 Gy or more.

In contrast, the apoptosis rate was barely influenced by irradiation even though the general level was enhanced in comparison to T cells or PBL. Still, an elevation plateau was detected following exposure of B cells to intermediate dose (0.3–1.0 Gy) of radiation. This suggested that B cells rapidly lost their membrane integrity and underwent secondary necrosis. This assumption was supported by the fact that the percentage of apoptotic cells declined with time but the percentage of of secondary necrotic cells increased. Furthermore, as already described for PBL and T cells, a decrease in apoptosis in favor of necrosis following exposure to very high radiation doses was observed for B cells (Figure 4B,C).

In direct comparison to T cells, the survival rates of B cells were similar but lower in all cases, showing the B cells were also more radiosensitive with regard to cell death induction. Even though secondary necrosis of B cells also increased over time in the nonirradiated controls, the impact of radiation on it was still obvious after exposure to a single dose of 0.3 Gy or higher.

### 2.4. Forms of Cell Death in NK Cells Following Radiation Exposure

**Figure 5:**
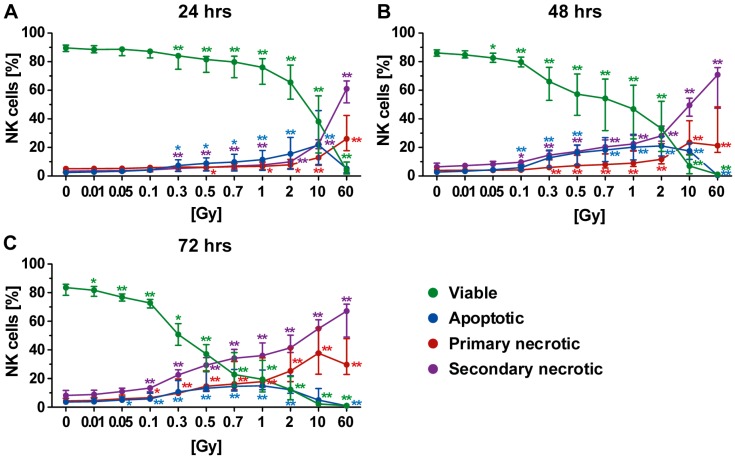
Forms of cell death in NK cells at different time points after irradiation. (**A**–**C**) NK cells were the most radiosensitive cells of all investigated human immune cells of the peripheral blood. The viability (green) already decreased after exposure to very low single dose of radiation. The most prominent type of cell death was secondary necrosis (violet), except at the early time point (24 h after radiation exposure) at which apoptosis (blue) had a higher impact. The colored dots represent the percentage distribution of viable (green), apoptotic (blue), primary (red), or secondary necrotic (violet) NK cells as determined by AxPI staining and flow cytometry at (**A**) 24, (**B**) 48, or (**C**) 72 h after irradiation. Each data point represents the median (±IQR) from eight independent experiments from four different donors. Data points have been connected by lines to improve visual clarity. Statistical analyses were performed against the corresponding nonirradiated control (0 Gy) using the Mann–Whitney *U* test (* *p* < 0.05; ** *p* < 0.01).

The NK cells were the most radiosensitive cells of the PBL with regard to cell death induction. A lowered viability was even observed 72 h after exposure of NK cells to a single dose of 0.01 Gy (Figure 5C). However, cell death rates were very small at this very low dose and became more pronounced starting from a single dose of 0.3 Gy (Figure 5A–C).

Twenty-four hours post irradiation, apoptotic cell death was the prominent form of cell death, which could already be observed following irradiation of NK cells with 0.3 Gy (Figure 5A). After exposure of NK cells to a high single dose (10 or 60 Gy), apoptosis decreased and necrosis increased, as already observed for T and B cells. Forty-eight hours after irradiation, the percentage of apoptotic cells was further increased (Figure 5B) but remained similar for the rest of the observation period (Figure 5C). At the two later time points (48 and 72 h), secondary necrosis was the major form of NK cell death (Figure 5B,C), as already observed for B cells (Figure 4B,C). These secondary necrotic cells very likely resulted from initially apoptotic cells. An intermediate state of both the apoptotic and secondary necrotic B cells was observed 48 h after radiation exposure (Figure 5B). Furthermore, a dose- and time-dependent increase in primary necrotic B cells was seen (Figure 5A–C). Regarding culturing conditions, like for T cells, only a marginal impact on NK cell death induction was found.

### 2.5. Forms of Cell Death in Monocytes Following Radiation Exposure

**Figure 6:**
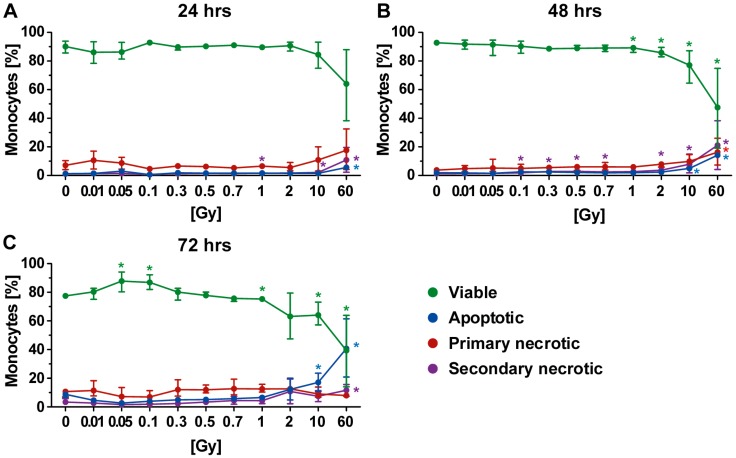
Forms of cell death in monocytes at different time points after irradiation. (**A**–**C**) Decreased viability of monocytes was only observed following exposure to a high single dose of irradiation. The colored dots represent the percentage distribution of viable (green), apoptotic (blue), primary (red), or secondary necrotic (violet) monocytes as determined by AxPI staining and flow cytometry at (**A**) 24, (**B**) 48, or (**C**) 72 h after irradiation. Each data point represents the median (±IQR) from four independent experiments of two different donors. Data points have been connected by lines to improve visual clarity. Statistical analyses were performed against the corresponding non-irradiated control (0 Gy) using the Mann–Whitney *U* test (* *p* < 0.05; ** *p* < 0.01).

In comparison to PBL, monocytes were identified to be very resistant to radiation (Figure 6). Even at later time points and after exposure to the very high single dose of 60 Gy, more than 40% of the monocytes were still viable (Figure 6B,C). Following radiation with a high single dose (10 or 60 Gy), a mixture of apoptosis and necrosis was observed.

## 3. Discussion

Even though the radiosensitivity of immune cells has already been examined in the past (summarized in Reference [29]), limited information is available on the sensitivity of human immune cells regarding radiation-induced forms of cell death. This is mostly because most of the already published work used Comet Assay, colony formation, and/or survival fractions as read-out system. This can be ascribed to the fact that radiation responses were exclusively thought to be related to DNA damage and consecutive responses—the so-called targeted effects of radiation. However, during the last decade, it has become evident that non-DNA-targeted effects of ionizing radiation, including genomic instability and a variety of bystander effects including immune modulations, do strongly contribute to radiation responses (summarized in References [30,31]). Therefore, the time has come to determine radiosensitivity of immune cells regarding induction of distinct forms of cell death as the latter have different immunological consequences. While apoptosis is non- or even anti-inflammatory, necrosis activates the immune system [32].

In this study, we performed detailed analysis of induction of apoptosis, secondary necrosis, and primary necrosis in key human cells of the innate and adaptive immune system. Furthermore, a wide spectrum of radiation dose was covered. As humans are constantly exposed to external radiation caused by natural background sources and diagnostic procedures, low doses of ≤0.1 Gy have to be considered for reasons of radiation protection [33]. The intermediate dose range (0.3–1.0 Gy) takes into account consequences of LDRT of benign inflammatory and/or erosive diseases [34]. The upper range (>1.0 Gy) considers effects of HDRT of malign diseases. For the latter, single dose per fraction of about 2 Gy is used, with cumulative doses of about 60 Gy [35]. Even though fractionated irradiation is used in the clinics, in this study, we focused an ex vivo/in vitro model system on irradiation with a single dose. In both benign and malign disease therapies, not all immune cells that circulate are hit by irradiation every fraction [24]. To get first indication about the general radiosensitivity of immune cells with regards to cell death induction, ex vivo experiments with single dose per fraction over a wide dose range should be a first approach. Additionally, as some immune cell types, such as B cells, undergo cell death due to culturing conditions over time (Figure 4), radiation-related effects are harder to judge with in vitro experiments, which last over a week or even longer as is the case when performing experiments with repeated fractionated irradiation. This is most prominent for granulocytes, which very rapidly undergo spontaneous cell death ex vivo [18] (Figure S1).

We did not find any impact of low-dose radiation exposure (single dose ≤0.1 Gy) on the examined human immune cells of the peripheral blood regarding cell death induction. The exception here was for NK cells, where a slight increase in both apoptotic and secondary necrotic cells was observed at later time points after radiation exposure (Figure 5). One could argue that, for example, even multiple chest X-ray examinations with a single dose of about 0.02–0.04 mSv do not induce cell death in key human immune cells.

However, the impact of functional alterations should be addressed in more detail in future examinations. Modulations of activation markers on immune in the peripheral blood after radon spa have recently been described [11]. Interestingly, Heylmann et al. discovered in 2018 that stimulated T cells were less radiosensitive than nonstimulated ones [36]. They also determined radiation-induced cell death with AxPI -staining. They found that T cells mainly underwent apoptosis following irradiation with 0.5 or 1.0 Gy. However, they did not distinguish between apoptotic cells with intact cell membrane and secondary necrotic/late apoptotic ones with disturbed membrane integrity. Taking this into account, our findings are in accordance with theirs as it showed that secondary necrosis was a prominent form of cell death after exposure of T cells of the peripheral blood to 0.5 or 1.0 Gy in our analyses (Figure 3). Secondary necrotic cells are apoptotic cells that have lost their membrane integrity at any time point during apoptosis. As shown in Figure 2, cells with subG1 DNA content were either apoptotic or secondary necrotic. In contrast, primary necrotic cells had lost their membrane integrity but still had full DNA content of a viable cell [18]. After 60 Gy of irradiation, cells that underwent apoptosis quickly lost their membrane integrity as seen by the complementary increase in secondary necrotic cells. Furthermore, at a high single dose, primary necrotic cells were induced directly as proven by the fact there were less cells with degraded DNA when compared to 10 Gy of irradiation (Figure 2). Heylmann et al. looked deeper in the mechanism and revealed that ATM serine/threonine kinase was the key regulator of the high radiosensitivity of resting T cells. This highlights that DNA damage responses and induction of distinct forms of cell death might be highly interconnected; this should be studied in more detail in the future [37]. Pugh et al. discovered that the chromatin state was a determinant of immediate efficient repair of DNA damage in T cells. This might also explain distinct differences in radiosensitivity of T cell subsets [38]. The low apoptosis rate of T cells observed in our study should not lead to assumption that T cells are not sensitive to lethal effects of ionizing radiation. Nakamura et al. demonstrated with dose-survival curves that T cells were highly radiosensitive [39]. We confirmed that approximately 50% of T cells died after exposure to a single dose of 2Gy (Figure 3B). However, these immune cells did lose their membrane integrity very quickly and were primary and secondary necrotic. One has to always carefully consider the read-out system on which analyses for radiosensitivity testing are based [40]. Even though radiosensitivity regarding the survival curves is similar of CD4+ and CD8+ T cells [39], future work should also examine whether the forms of cell death differ between different subsets of T cells, such as CD4+, CD8+, and regulatory T cells.

In chronic inflammatory diseases, such as rheumatoid arthritis, a hyperactivity of CD4^+^ T helper cells has also been described to play a crucial role [41]. Therefore, elimination of those cells by LDRT could be an efficient way of restoring a balance in a chronically inflamed tissue. However, necrosis induction of T cells by radiation in the intermediate dose range should also be taken into account. Even primary necrotic cells can be removed by macrophages in an anti-inflammatory manner [42], again potentially contributing to beneficial effects of LDRT [7].

Concerning the dose range of ionizing radiation, which is applied for LDRT of benign degenerative and/or inflammatory diseases, more impact of radiation on cell death induction of immune cells was observed. Here, monocytes were the most radioresistant immune cells. Even though Kaina and colleagues demonstrated that monocytes are more responsive to cell death induction by ROS when compared to macrophages and dendritic cells, the amount of ROS produced by a single dose up to 2 Gy had only minor impact on the viability of monocytes [43]. This is in accordance with our examinations, which showed that only irradiation with a high single dose of 10 or 60 Gy induced enhanced cell death in monocytes (Figure 6). One has to emphasize that induction of apoptosis in monocytes therefore does not contribute to the observed anti-inflammatory effects of LDRT, as described earlier for PBL [44]. However, previous examinations from our group have revealed that LDRT influences the functionality of monocytic cells. For instance, Frischholz et al. demonstrated a decreased secretion of the inflammatory cytokine IL-1beta by macrophages of radiosensitive Balb/c mice following LDRT [45]. As monocytes in the peripheral blood, tumor-infiltrating monocytes might also undergo a mixture of apoptotic and necrotic cell death during the treatment with HDRT. However, this could be beneficial as tumor-associated macrophages that do differentiate from monocytes foster, rather than suppress, tumor growth [46]. Still, a major part of monocytes in our analyses even survived doses up to 60 Gy (65% after 24 h to 40% after 72 h). This makes monocytes that are more radioresistant ideal target for combination of HDRT with immunotherapy [47].

We further investigated the radiosensitivity of granulocates, but these polymorphonuclear innate immune cells (PMN) died quickly due to in vitro culturing conditions, resulting in less than 30% viable cells in the nonirradiated controls within the first 12 h as already previously described [18,48,49]. However, we observed that the remaining PMN did not show signs of further cell death induction up to irradiation with 60 Gy (Figure S1). Thus, the more radioresistant myeloid cells might have a particular cell repair mechanism or ability of maintaining integrity that is worthwhile for further examination.

B and NK cells were more radiosensitive when compared to T cells regarding cell death induction, with NK cells being the most sensitive ones. Twenty-four hours after irradiation, NK and B cells showed decreases in viability by 10–15% (0.7 Gy). Later, this decrease reached 40–50% (0.7 Gy; 48 h) or even more than 70% (0.7 Gy; 72 h). Our investigations revealed that human immune cells do not only differ in their radiosensitivity but also in the way they die. T cells displayed high amounts of primary and secondary necrotic cells following radiation exposure, while more apoptosis was observed in B and NK cells.

The third investigated dose range (≥2.0 Gy) represented the HDRT. Here, the cytotoxic potential of ionizing radiation is well known and even desired for elimination of tumor cells within the radiation field. Yet, it is unclear how invading immune cells are affected and whether standard fractionation schemes impede the development of effective antitumor immunity (summarized in Reference [3]). As T cell viability was strongly reduced within this dose range in our study, one has to thoroughly reflect re-irradiation time points of the tumor because beneficial T-cell-mediated antitumor responses within the tumor might be destroyed [22]. T cells are ideal candidates for immunotherapy in combination with radiotherapy [50], but the fractionation schemes should again be carefully selected. Furthermore, an in-depth investigation of the subsets might be worthwhile, especially as it already has been reported that regulatory T cells are more radioresistant than other T cells [27,29]. For beneficial anticancer therapy, regulatory T cells within the tumor should be killed, while cytotoxic CD8+ ones should be protected [51].

In vivo data on radiosensitivity of immune cells have already been collected by analyzing, for example, splenocytes of total-body exposed mice. However, cell death analyses in these studies did not differ between apoptosis and necrosis. Regarding apoptosis as detected by TdT-mediated dUTP-biotin nick end labeling (TUNEL) assay, B cells have been found to be the most radiosensitive cells following exposure to 0.5 or 2.0 Gy [27]. This is accordance with our results as apoptosis was one prominent form of cell death in human B cells after exposure to a single dose of 0.3 Gy or higher in our study (Figure 4).

In humans, the bone marrow and the small bowel are generally the two major organs injured by radiation [52]. Total body irradiation is also used for conditioning of tumor patients for allogeneic bone marrow transplantation [53]. This highlights that immune cells are sensitive to radiation. Our results confirmed that at higher single dose per fraction (≥2 Gy), immune cells died, not only by apoptosis but also by necrosis. Former studies on lymphocyte populations in the blood of patients with nonhematological tumors undergoing local radiotherapy have revealed B cells to be the most radiosensitive cells as determined by reduced counts in the peripheral blood. Here again, no significant differences between T cell subsets were observed [54]. We also identified that B cells of the peripheral blood were less resistant to radiation in comparison to T cells. Future analyses should take into account that cell death responses following radiation exposure might, under distinct microenvironmental conditions, be dependent on age and sex [55] as well as on the basal inflammatory state [7,8]. Our ex vivo/in vitro studies should also be considered as the first helpful basis to optimize (individualize) radiotherapy in multimodal settings, including for the definition of suited time points for further inclusion of immunotherapies or for reducing the cumulative dose of radiation.

## 4. Materials and Methods

### 4.1. Cell Separation and Treatment

Peripheral blood samples were obtained from voluntary normal healthy donors. This was approved by the ethics committee of the Bayerische Landesärztekammer (#12131; 06.03.2013) in accordance with the principles described in the current version of the Declaration of Helsinki. All donors accepted and provided written informed consent. The blood withdrawals were performed using 20 mL syringes (Becton, Dickinson & Company, Franklin Lakes, NJ, USA) containing 12.5 U/mL Heparin (B. Braun, Melsungen, Germany). The first step in cell isolation from fresh human whole blood samples was Ficoll (Bio-Rad Medical Diagnostic GmbH, Dreieich, Germany) density separation into a polymorphonuclear cells (PMN) and a peripheral blood mononuclear cells) fraction according to manufactures protocol (PBMC).

To obtain pure PMN, the erythrocytes were removed from PMN fraction by lysis with deionized water (ddH_2_O). Therefore, 9 mL of ddH_2_O were added and shaken for 20 s followed by re-buffering with 1 mL of 10-times concentrated phosphate-buffered saline (PBS, pH 7.4; Life Technologies, Carlsbad, CA, USA). The PBL and monocytes were obtained by removal of monocytes from the PBMC fraction by adhesion to cell culture plate (Greiner bio-one, Frickenhausen, Germany) bottom for 1 h. The purity of PMN, PBL, and monocytes was verified by flow cytometry (Figure S2). The purification of T cells, B cells, and NK cells from the PBMC faction was conducted by positive magnetic bead separation (Miltenyi Biotec GmbH, Bergisch Gladbach, Germany) according to the manufacturer’s protocol. Therefore, anti-CD3 (T cells), anti CD19 (B cells), or CD56-beads (NK cells) were used. To improve purity, the labeled cells were passed twice through the columns. The purity of the isolated immune cells was verified by flow cytometry (Figure S3).

Finally, the isolated cells were washed twice with PBS (Sigma Life Science, St. Louis, MO, USA) and seeded into 24-well cell culture plates (1 × 10^6^ cells/well; Greiner bio-one, Frickenhausen, Germany) in RPMI-1640 medium (Sigma Life Science) containing 10% of heat-inactivated FBS (FBS Superior standardized, Biochrom, Berlin, Germany), penicillin (100 U/mL; Life Technologies, Darmstadt, Germany), and streptomycin (100 µg/mL; Life Technologies). 

After 30 min of incubation (37 °C, 5% CO_2,_ 95% humidity), the cells were irradiated with various single doses ranging from 0.01–60 Gy using an X-ray tube (100–120 kV, varying time; ISOVOLT Titan, General Electric, Boston, MA, USA). Consecutively, the cells were incubated under standard conditions (37 °C, 5% CO_2_, 95% humidity) and harvested after 24, 48, or 72 h. The blood of at least two different volunteers was used for the analyses and the experiments were performed at least twice with cells of each volunteer. Furthermore, blood of at least one male and one female donor was used. The average age of the donors was 27.18 years.

### 4.2. Cell Staining and Data Acquisition

Apoptotic (AxV+, PI−), primary (AxV+, PI++), and secondary necrotic (AxV+, PI+) cells were determined by flow cytometry using AxPI staining, which combines FITC-labeled AnnexinV (0.1 µg/mL; life technologies, GeneArt, Regensburg, Germany) and PI (0.2 µg/mL; Sigma Life Science) (Figure 1B). Viable cells were defined as being negative for both AxV and PI staining. The complementary value of the total percentage of dying and dead cells (apoptotic, primary, and secondary necrotic cells) represented the viable cell fraction. In addition, PI staining in presence of the detergent triton (PIT staining) was performed to determine the subG1 DNA content of apoptotic and secondary necrotic cells [56]. For this, cell membranes were first disintegrated using triton (0.05% (*v*/*v*); Sigma Aldrich, St. Louis, MO, USA), and DNA was then stained by PI (5 µg/mL).

Following incubation for 30 min at 4 °C in the dark, the AxPI-stained cells were measured using the Gallios flow cytometer (Beckman Coulter, Brea, CA, USA), and the subG1 DNA content determination was performed using the EPICS XL flow cytometer (Beckman Coulter). Prior to measurement, optimal cytometer settings and compensation values were determined using unstained and single-stained controls.

### 4.3. Data Analysis

The data obtained by flow cytometry were analyzed using the Kaluza analysis software (v. 1.3; Beckman Coulter) followed by calculation of the mean values using MS Excel (version 14.07214.5000 (32-Bit), Microsoft Corporation, Redmond, WA, USA). The statistical analysis was performed and graphs were created using GraphPad Prism (v. 5.04; GraphPad Software Inc., La Jolla, CA, USA). Irradiated cells were compared to their nonirradiated controls of the same time point using the Mann–Whitney *U* test.

## Supplementary Materials

Supplementary materials can be found at http://www.mdpi.com/1422-0067/19/11/3574/s1, Figure S1: Cell death forms of granulocytes at different time points after irradiation, Figure S2: Purity of immune cell populations after density separation of peripheral blood polymorphonuclear cells (PMNs, granulocytes) and peripheral blood mononuclear cells (PBMCs) from fresh human whole blood, Figure S3: Purity of immune cell populations after magnetic separation.

## Abbreviations

RT	Radiotherapy
HDRT	high-dose radiation therapy
LDRT	low-dose radiation therapy
PBMC	peripheral blood mononuclear cells
PBL	peripheral blood lymphoid cells
TREGs	regulatory T cells
PMN	polymorph nuclear cells
NK cells	natural killer cells
PIT staining	propidium iodide in the presence of triton staining
AxPI staining	FITC-labeled AnnexinV and propidium iodide staining
IQR	interquartile range
DNA	deoxyribonucleic acid
DSB	double strand break
ROS	reactive oxygen species
