# Supplementary material for: Clinically Relevant Radiation Exposure Differentially Impacts Forms of Cell Death in Human Cells of the Innate and Adaptive Immune System
Source: Int J Mol Sci. 2018 Nov 13;19(11):3574. doi: 10.3390/ijms19113574 (PMC6274975; doi:10.3390/ijms19113574)
Supplement: Supplementary file 1 [file ijms-19-03574-s001.pdf]

International Journal of
Molecular Sciences

MDPI

Research Paper

Clinically relevant radiation exposure differentially impacts on cell death forms of human cells of the innate and adaptive immune system

# - Supplementary material -

Sylvia E. Falcke, Paul F. Rühle, Lisa Deloch, Rainer Fietkau, Benjamin Frey, and Udo S. Gaipl

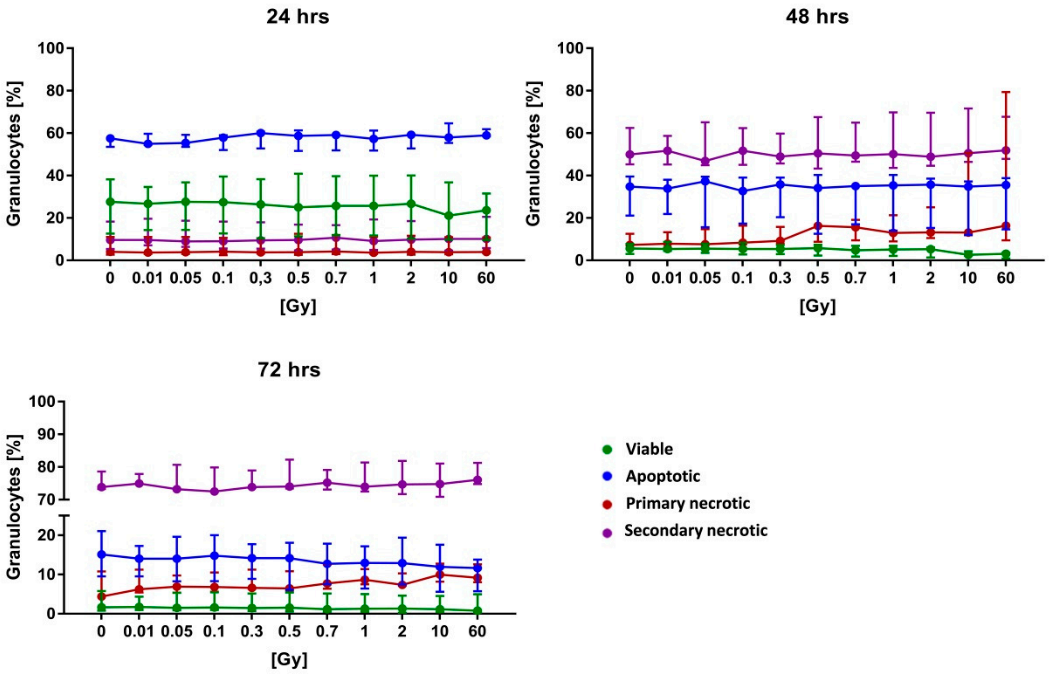

Supplementary Figure 1: Cell death forms of granulocytes at different time points after irradiation. (**A-C**) The colored dots represent the percentage distribution of viable (**green**), apoptotic (**blue**), primary (**red**) or secondary necrotic (**violet**) granulocytes as determined by AxPI-staining and flow cytometry at 24 (**A**), 48 (**B**) or 72 hours (**C**) after irradiation. Each data point represents the median (± IQR) from four independent experiments of two different donors. Data points were connected by lines to improve visual clarity. Statistical analyses were performed against the corresponding non-irradiated control (0 Gy) using the Mann Whitney U test.

© 2018 by the authors; licensee MDPI, Basel, Switzerland. This article is an open access article distributed under the terms and conditions of the Creative Commons by Attribution (CC-BY) license (http://creativecommons.org/licenses/by/4.0/).

Research Paper

Clinically relevant radiation exposure differentially impacts on cell death forms of human cells of the innate and adaptive immune system

# - Supplementary material -

Sylvia E. Falcke, Paul F. Rühle, Lisa Deloch, Rainer Fietkau, Benjamin Frey, and Udo S. Gaipl

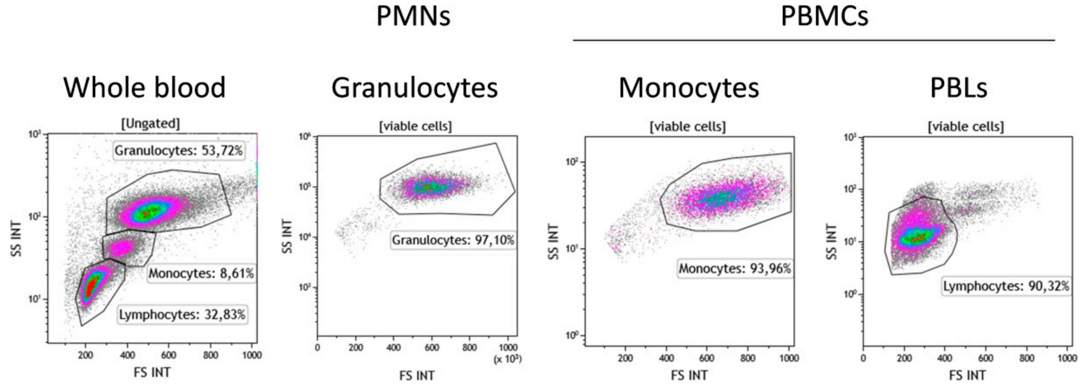

Supplementary Figure 2: Purity of immune cell populations after density separation of peripheral blood polymorphonuclear cells (PMNs, granulocytes) and peripheral blood mononuclear cells (PBMCs) from fresh human whole blood. Peripheral blood lymphocytes (PBLs) and monocytes were subsequently obtained by adhesion of monocytes to cell culture plates. The purity of PMNs used for the experiments was approximately 97% and that of monocytes and PBLs about 94% and 90%, respectively.

© 2018 by the authors; licensee MDPI, Basel, Switzerland. This article is an open access article distributed under the terms and conditions of the Creative Commons by Attribution (CC-BY) license (http://creativecommons.org/licenses/by/4.0/).

Research Paper

Clinically relevant radiation exposure differentially impacts on cell death forms of human cells of the innate and adaptive immune system

# - Supplementary material -

Sylvia E. Falcke, Paul F. Rühle, Lisa Deloch, Rainer Fietkau, Benjamin Frey, and Udo S. Gaipl

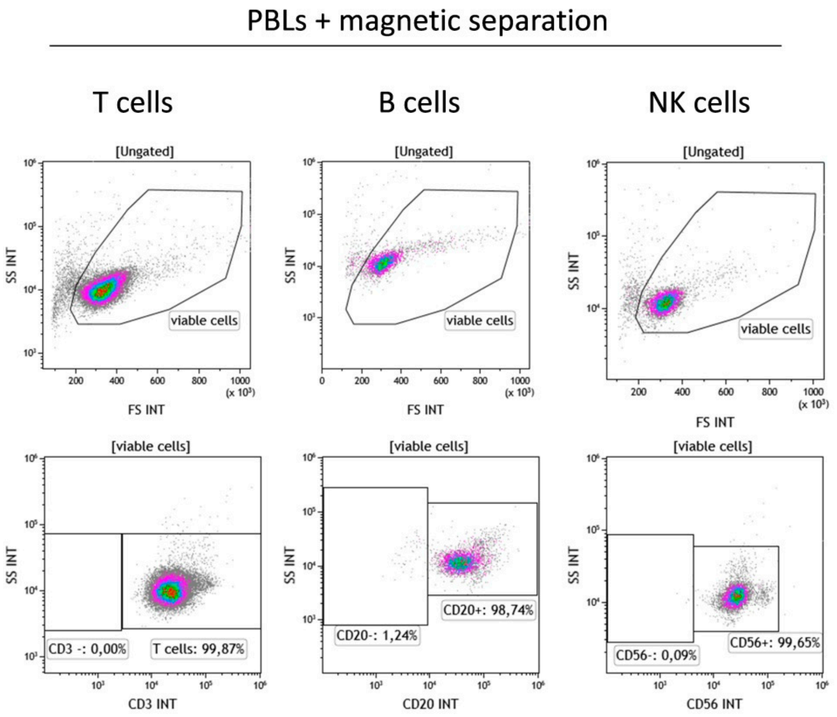

Supplementary Figure 3: Purity of immune cell populations after magnetic separation. After the separation the cells remained viable (upper line). The purity of T cells (CD3+), B cells (CD20+) and NK cells (CD56+) was about 99% in all cases (lower line). Isotype controls were used to determine positive and negative gates for the respective immune cell types.

© 2018 by the authors; licensee MDPI, Basel, Switzerland. This article is an open access article distributed under the terms and conditions of the Creative Commons by Attribution (CC-BY) license (http://creativecommons.org/licenses/by/4.0/).
